# Validating an Evaporative Calibrator for Gaseous Oxidized Mercury

**DOI:** 10.3390/s21072501

**Published:** 2021-04-03

**Authors:** Jan Gačnik, Igor Živković, Sergio Ribeiro Guevara, Radojko Jaćimović, Jože Kotnik, Milena Horvat

**Affiliations:** 1 Jožef Stefan International Postgraduate School, Jamova Cesta 39, 1000 Ljubljana, Slovenia; jan.gacnik@ijs.si; 2 Department of Environmental Sciences, Jožef Stefan Institute, Jamova Cesta 39, 1000 Ljubljana, Slovenia; igor.zivkovic@ijs.si (I.Ž.); radojko.jacimovic@ijs.si (R.J.); joze.kotnik@ijs.si (J.K.); 3 Laboratorio de Análisis por Activación Neutrónica, Centro Atómico Bariloche, Av. Bustillo km 9.5, Bariloche 8400, Argentina; ribeiro@cab.cnea.gov.ar

**Keywords:** gaseous oxidized mercury, traceability, calibration, ^197^Hg radiotracer

## Abstract

Understanding atmospheric mercury chemistry is the key for explaining the biogeochemical cycle of mercury and for improving the predictive capability of computational models. Increased efforts are being made to ensure comparable Hg speciation measurements in the air through establishing metrological traceability. While traceability for elemental mercury has been recently set, this is by no means the case for gaseous oxidized mercury (GOM). Since a calibration unit suitable for traceable GOM calibrations based on evaporation of HgCl_2_ solution was recently developed, the purpose of our work was to extensively evaluate its performance. A highly specific and sensitive ^197^Hg radiotracer was used for validation over a wide range of concentrations. By comparing experimental and calculated values, we obtained recoveries for the calibration unit. The average recoveries ranged from 88.5% for 1178 ng m^−3^ HgCl_2_ gas concentration to 39.4% for 5.90 ng m^−3^ HgCl_2_ gas concentration. The losses were due to the adsorption of oxidized Hg on the inner walls of the calibrator and tubing. An adsorption isotherm was applied to estimate adsorption enthalpy (ΔH_ads_); a ΔH_ads_ value of −12.33 kJ mol^−1^ was obtained, suggesting exothermal adsorption. The results of the calibrator performance evaluation suggest that a newly developed calibration unit is only suitable for concentrations of HgCl_2_ higher than 1 µg m^−3^. The concentration dependence of recoveries prevents the system from being used for calibration of instruments for ambient GOM measurements. Moreover, the previously assessed uncertainty of this unit at µg m^−3^ level (2.0%, k = 2) was re-evaluated by including uncertainty related to recovery and was found to be 4.1%, k = 2. Calibrator performance was also evaluated for HgBr_2_ gas calibration; the recoveries were much lower for HgBr_2_ gas than for HgCl_2_ gas even at a high HgBr_2_ gas concentration (>1 µg m^−3^). As HgBr_2_ is often used as a proxy for various atmospheric HgBr species, the suitability of the unit for such calibration must be further developed.

## 1. Introduction

Mercury is present in the atmosphere in different forms as a result of anthropogenic activities and natural processes. When in air, mercury can be carried long distances across the hemisphere and deposited into terrestrial and aquatic environments, where it is taken into the food web or re-emitted into air [1]. Atmospheric Hg fractions are operationally defined as gaseous elemental mercury (GEM, Hg^0^), gaseous oxidized mercury (GOM, Hg^2+^), particulate-bound mercury (PBM, Hg-p), and total gaseous mercury (TGM). Since the atmosphere is the major pathway for global Hg transport, understanding the atmospheric Hg cycle is of great importance. Hg speciation is, therefore, a critical parameter of understanding the Hg atmospheric cycle [2].

Even though GEM is the most abundant atmospheric Hg form, PBM and especially GOM are also crucial in the atmospheric Hg cycle, as they serve as atmospheric mercury sink [2,3,4]. Since GOM and PBM are more soluble and have shorter lifetimes than GEM, the knowledge of their wet and dry deposition and their occurring oxidation patterns is required. Method calibration, quantification of interferences and fundamental research on the speciation and behavior of these species are also still needed [4,5].

The results obtained for oxidized mercury species in the air are largely dependent on the method used for separating different mercury species/fractions [6,7]. Moreover, due to the absence of common calibration of the instruments, the results cannot be directly compared. Reliable comparisons of such data present a great challenge for researchers [2,7,8,9]. Metrological traceability of atmospheric Hg measurements needs to be ensured in order to achieve comparable data, starting at the traceable calibration of the analytical instrument. The International Vocabulary of Metrology (VIM) defines metrological traceability as a “property of a measurement result whereby the result can be related to a reference through a documented unbroken chain of calibrations, each contributing to the measurement uncertainty” [10]. Recently, there has been progress in ensuring SI traceability of GEM calibrations. SI traceability was achieved by Ent et al. [11], with a mercury vapor generator based upon an improved diffusion method that exploited gravimetric analysis to achieve traceability to the SI unit (mass) [11]. Živković et al. [12] loaded iodinated activated carbon (AC) traps by reducing NIST SRM 3133 and by directly spiking the said SRM (Standard Reference Material^®^). The results showed that traceable Hg^0^ calibration of an iodinated AC trap can be achieved by direct SRM spiking. Quétel et al. used calibration based upon isotope dilution ICP-MS in the liquid phase, combined with automated handling of the air samples, to achieve SI traceability [13,14]. Isotope dilution ICP-MS was also used to calibrate the GEM generator developed by National Institute of Standards and Technology (NIST). NIST SRM 3133 (Mercury Standard Solution) was used for calibrating the ICP-MS method. Since NIST SRM 3133 is traceable to SI units, an unbroken chain of traceability to SI units was achieved [15]. Srivastava et al. [16] applied a high-resolution absorption spectroscopy method to achieve SI traceability for the GEM measurement. SI traceability was achieved by modeling the observations from the first principles (ab initio) and from the overall low measurement uncertainty [16]. Even though SI traceable GEM calibrations are now available, bell-jar calibration with mercury vapor is still widely used [17]. The empirical equation, which describes the relationship between the saturated Hg vapor and the temperature, is not universally agreed upon, which questions the comparability of the obtained data [8,17,18,19,20].

To date, the literature offers no SI traceable calibrations for GOM. In the last decade, a system produced by Tekran^®^ (Tekran 2537A) has been the most widely used one for Hg speciation [21,22,23,24]. It is usually calibrated with the elemental Hg obtained from a bell-jar apparatus or with its internal calibration, which is performed using a mercury permeation tube that is located in a temperature-controlled chamber within the instrument itself [25]. In addition to lacking traceable calibrations, atmospheric Hg speciation systems are known to be dependent on the sampling method, meteorological conditions, and air quality (i.e., high humidity and high ozone concentration) [9,26,27].

Direct calibration with the gaseous Hg^2+^ species instead of Hg^0^ is needed to obtain reliable measurements. Lyman et al. [28] developed a novel calibration unit for Hg atmospheric species using a special design of a permeation oven. The newly developed calibrator achieved stable permeation rates that were high enough to achieve gravimetric verification while emitting relatively high amounts of Hg^0^ together with HgBr_2_ and HgCl_2_. These amounts were not quantitatively determined; hence, an independent, traceable, and reliable source of Hg compounds was not completely achieved [28]. Recently, efforts have been made to validate the permeation calibration via gravimetric methods. The permeation rates measured by the Tekran 1130 unit, dual-channel system, and gravimetric measurements were not always in agreement, so traceability to the mass has not yet been established [29]. A novel calibration method for the HgBr_2_ species was implemented by McClure et al. [30]; the authors constructed a permeation tube with HgBr_2_ crystals inside the tube. Schaedlich et al. [31] patented a calibration system (Hovacal^®^, IAS GmbH, Oberusel, Germany) that generates humidified gas from the chosen solution. For the purpose of GOM calibration, HgCl_2_ solutions of different concentrations can be used [31]. The system requires precise control of the temperature, as well as the flowrate, and is additionally prone to vibrations–all of which are hard to control in field measurements. Moreover, Hg^0^ impurities are a known problem, as they introduce uncertainty to such calibration [32]. All GOM generation systems discussed in the introduction are shown and compared in Table 1.

Recently, Optoseven Ltd., together with VTT Ltd., developed a portable gas generator suitable for generating gaseous Hg^2+^ species within the framework of the MercOx project (MercOx 16ENV01, Metrology for oxidized mercury). An evaporation method was applied to generate oxidized mercury gas. HgCl_2_ solution of a known concentration is injected into the flow of the carrier gas (e.g., nitrogen) and is evaporated in an evaporation chamber at over 120 °C to form HgCl_2_ gas. More information regarding the calibration unit is available elsewhere [33]. The calibration unit was previously tested for >1000 ng m^−3^ HgCl_2_ gas concentrations. Although the flue gas GOM concentrations are in the range of 1000 ng m^−3^ [34,35], the ambient GOM concentrations range from 1–300 pg m^−3^ [36,37,38], and calibrators also need to be validated for such concentration levels. Petrov et al. [39] evaluated the performance of the calibration unit for the ng m^−3^ to µg m^−3^ concentration range and observed satisfactory results. While the authors obtained great stability, response time, and linearity, the validity of the output still has to be evaluated, especially for low gas concentrations [39]. To enable low concentration validation, stable [40,41,42,43] and radioactive [44,45,46,47,48] mercury isotopes can be used. While stable mercury isotopes and isotope dilution mass spectrometry (IDMS) are more commonly used, radioactive Hg isotopes have proven to be advantageous in situations where contamination and detection limits are problematic. Low detection limits can be achieved with radioactive ^197^Hg due to the high specific activity, which can be obtained by the irradiation of ^196^Hg-enriched elemental mercury with thermal neutrons (thermal (n,γ) cross section of 3080 barns for ^196^Hg, one of the highest of all nuclides [49]). To the best of our knowledge, no work has been done on validating the calibration unit for low Hg concentrations. Our objective was to test the calibration unit over longer periods of constant operation and different concentration levels. Speciation measurements of Hg^2+^ and Hg^0^ would also be addressed together with the adsorption of Hg^2+^ on the inner parts of the calibration unit and tubing.

## 2. Materials and Methods

### 2.1. Chemicals and Instruments

Chemicals used in our work: 95%–97% H_2_SO_4_ (for analysis, Merck, Darmstadt, Germany), 65% HNO_3_ (for analysis, Supelco, Darmstadt, Germany), 30% HCl (suprapur, Merck, Darmstadt, Germany), 47% HBr (for analysis, Merck, Darmstadt, Germany), KCl (suprapur, Merck, Darmstadt, Germany), KMnO_4_ (for analysis, max. 0.000005% Hg, Merck, Darmstadt, Germany), HgCl_2_ (≥99.5% purity, Sigma-Aldrich, Darmstadt, Germany), NIST SRM 3133: Mercury (Hg) Standard Solution (National Institute of Standards and Technology, Gaithersburg, MD, USA), ^196^Hg enriched elemental Hg (enriched to 51.58% ^196^Hg, Isoflex, San Francisco, CA, USA), and Type I purified water (electrical resistivity 18.2 MΩ cm; Milli-Q water, Merck, Darmstadt, Germany). Instruments used in our work: high-purity germanium (HPGe) well-type detector (model GCW6023/S, Canberra Industries Inc., Meriden, CT, USA), cold vapor atomic absorption spectrometer (Model Hg-201 Semi-Automated Mercury Analyzer, Sanso Seisakusho Co., Ltd., Tokyo, Japan), and a liquid evaporative generator for oxidized mercury (Optoseven Ltd. & VTT Ltd., Espoo, Finland).

**Figure 1:**
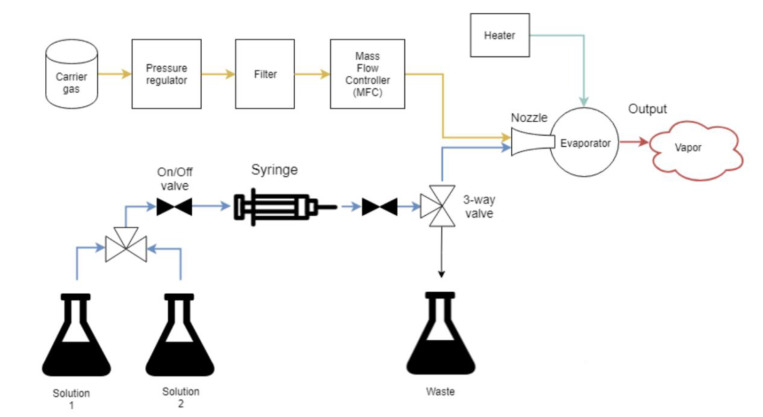
The flowchart of the liquid evaporative generator developed by Optoseven Ltd. and VTT Ltd.

The Optoseven liquid evaporative generator is a portable calibration unit for oxidized mercury. The flowchart of the Optoseven liquid evaporative generator is shown in Figure 1. First, the HgCl_2_ calibration solution is prepared by dissolving a known amount of Hg^2+^ salt and by adding an adequate amount of HNO_3_ and HCl (0.1% *v*/*v* for both acids). The HgCl_2_ solution is pumped by an automatic syringe pump, which can produce precise and low liquid flow rates. The solution is mixed with carrier gas (dry, clean gas, e.g., nitrogen) in the evaporator. The thermal mass flow controller ensures a known mass flow rate of the carrier gas into the evaporator. All parts of the evaporator are covered with a chemically inert polytetrafluoroethylene (PTFE). In the evaporator, the nozzle mixes the carrier gas and the HgCl_2_ solution; it then sprays the mixture into the evaporation chamber, which is kept at a temperature of >120 °C. Here, the HgCl_2_ vapor is formed, which is the final output of the generator [33].

The output HgCl_2_ gas concentration is calculated by applying Equations (1) and (2).

(1) $$c_{HgCl_{2}}=\frac{c_{sol}\;Q_{sol}}{Q_{gas}+Q_{w}}$$

(2) $$Q_{w}=\frac{Q_{sol}\;R\;T\;}{M_{H_{2}O}\;p}$$

where:

*CHgCl_2_* is the concentration of HgCl_2_ in the output gas [ng m^−3^],
*C_sol_* is the concentration of HgCl_2_ in the calibration solution [ng L^−1^],
*Q_sol_* is the flowrate of HgCl_2_ in the calibration solution [mL min^−1^],
*Q_gas_* is the flowrate of the carrier gas [L min^−1^],
*Q_w_* is the flowrate of water [L min^−1^],
*R* is the gas constant [J K mol^−1^],
*T* is the temperature [K],
*M_H_*_2*O*_ is the molar mass of water [kg mol^−1^],
*p* is the pressure [Pa].

### 2.2. Production of the ^197^Hg Radiotracer

Elemental Hg, enriched to 51.58% in the ^196^Hg isotope (natural abundance is 0.15%), was used for production of the ^197^Hg radiotracer. Two milliliters of enriched ^196^Hg in 2% HNO_3_ acid (*v*/*v)* solution was sealed into a quartz ampoule and irradiated for 12 h in the central channel (CC) of the 250 kW TRIGA Mark II research reactor of the Jožef Stefan Institute (JSI), Ljubljana, Slovenia. The sample was irradiated in neutron flux of 10^13^ cm^−2^ s^−1^ and the ^197^Hg radionuclide was induced by ^196^Hg(n,γ)^197^Hg reaction. After irradiation, the Hg solution was transferred from the irradiated vial and diluted to appropriate Hg concentrations for subsequent experiments. In these experiments, solutions and gases of HgX_2_ (X = Cl^−^, Br^−^) species were used; their concentrations are given as Hg concentrations in the following paragraphs and not as HgX_2_ concentrations if not explicitly stated otherwise.

### 2.3. Determining ^197^Hg by Using a HPGe Detector

To obtain standards for gamma measurement, triplicates of a standard solution (8 mL, 2% HNO_3_ (*v*/*v*)) were transferred into glass vials. The standard solution was always diluted so that the obtained activity was similar to the activity of the measured sample. The activity (γ-rays and X-rays) of the standards in the vials was measured using a HPGe well-type detector.

Using Genie 2000 Gamma analysis software, the activity of ^197^Hg in the samples was determined by a peak area comparison of the characteristic γ-ray and X-ray emissions for ^197^Hg (*t*_1/2_ = 2.671 d, two doublet peaks: 67.0 + 68.8 keV and 77.3 + 78.1 keV). All of the obtained activities were re-calculated to a reference time by applying an equation derived from the exponential law of radioactive decay. The exact equations that were used for the calculation of the activity and recovery are available in the Supplementary Material [44,48,50].

The recoveries discussed in the results section were obtained by comparing the activities of the impinger solution samples (impinger solutions retained HgCl_2_ gas output of the calibrator–described in detail in the following sections) to the activities of the standard solutions (theoretical, 100% recovery values). To know exactly which activity level corresponded to which HgCl_2_ concentration, we connected the activities of the ^197^HgCl_2_ standard solutions to their concentration by CV-AAS measurement (calibration against NIST SRM 3133) [51]. The determined concentration for the stock HgCl_2_ solution was 93.3 µg mL^–1^ of Hg. CV-AAS measurement was performed before and after irradiation to show that there was no difference in the HgCl_2_ concentration before and after irradiation. From the HgCl_2_ solution concentration, we calculated the HgCl_2_ gas concentration in the calibrator output by applying Equations (1) and (2).

In our work, the solutions and gases of HgX_2_ (X = Cl^−^, Br^−^) species were used; their concentrations are given as Hg concentrations in the following paragraphs and not as HgX_2_ concentrations if not explicitly stated otherwise.

### 2.4. Calibrator Time Response Tests

#### 2.4.1. Calibrator Time Response Tests Using HgCl_2_ Gas

**Figure 2:**
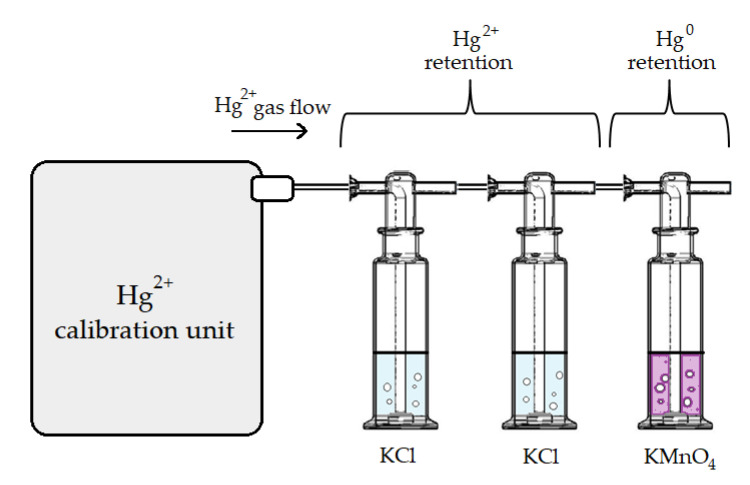
The three-impinger setup used for capturing the HgCl_2_ gas output of the calibration unit (objects not shown to scale).

To obtain a time-dependent result of the output from the calibrator, it was continuously operating for a period of four workdays (up to 75 h, including continuous operation overnight). Since continuous monitoring of the calibrator output was not possible due to the use of the ^197^Hg radiotracer, discrete measurements of the calibrator output were performed each day. In order to capture the calibrator output, a three-impinger setup was used as shown in Figure 2. This setup represents a considerable simplification of the Ontario Hydro (OH) method [52]. The setup was comprised of two KCl impingers (100 mL of 1 mol L^−1^ KCl solution) and one KMnO_4_ impinger (100 mL of 10% KMnO_4_ (*w*/*v*) in 20% H_2_SO_4_ (*v*/*v*) solution) downstream of the former. The KCl impingers were used for capturing Hg^2+^ while the Hg^0^ that passed through them was retained in the KMnO_4_ impinger [52].

The ^197^HgCl_2_ calibration solution for the Optoseven calibrator was prepared in 0.1% HCl (*v*/*v*) + 0.1% HNO_3_ (*v*/*v*) (in agreement with the work of Saxholm et al. [33]); the HgCl_2_ concentration depended on the concentration level that was tested, and it ranged from 0.467 ng mL^−1^ to 93.3 ng mL^−1^, expressed as Hg. The exact composition of the [HgCl_x_]^2−x^ species present in the calibration solution was calculated and will be presented in the results and discussion section.

The calibrator was tested for the following conditions: a ^197^HgCl_−_solution intake of 0.07 mL min^−1^, an N_2_ carrier gas flow of 5 L min^−1^, and an evaporation chamber temperature of 125 °C.

Prior to collecting the calibrator output, the calibrator was operated for 1 h for preconditioning in order to achieve a stable temperature in the evaporation chamber. The first discrete measurement taken during the first day was marked as *t* = 0. For a single discrete measurement, the output of the calibrator was collected for 10–20 min, depending on the activity of the radiotracer at the time of collection (a more decayed radiotracer required longer collection times). Eight milliliter aliquots of the impinger solutions were taken for gamma well measurement. Additionally, the ^197^Hg that was adsorbed into the impinger surfaces was thoroughly cleaned with 10 mL of 10% HNO_3_ (*v*/*v*) + 5% HCl (*v*/*v*) acid solution. Previous tests showed that this was the optimal acidic solution to quantitatively remove all oxidized Hg from the surfaces (results not shown). Eight milliliter aliquots of this washing solution were also taken for gamma measurement in the HPGe well-type detector.

During the 72 h of continuous calibrator operation, discrete measurements of the calibrator output were performed up to three times per workday. Some experiments had to be terminated before the 72 h had finished, due to the power supply malfunction of the calibrator. When the calibrator output was not being collected for discrete measurements, two KMnO_4_ impingers were used for ^197^Hg retention to avoid radioactive contamination.

#### 2.4.2. Calibrator Time Response Tests Using HgBr_2_ Gas

The preparation of standards, calculation of results, and experimental procedure were performed in the same way as described above. The only difference was that for the calibration solution, ^197^Hg^2+^ in 0.1 HBr (*v*/*v*) + 0.1 HNO_3_ (*v*/*v*) was used (two HgBr_2_ calibration solution concentrations were used, 5.60 and 93.3 ng mL^−1^ expressed as Hg).

### 2.5. Hg^2+^ Species Adsorption Experiments

**Figure 3:**
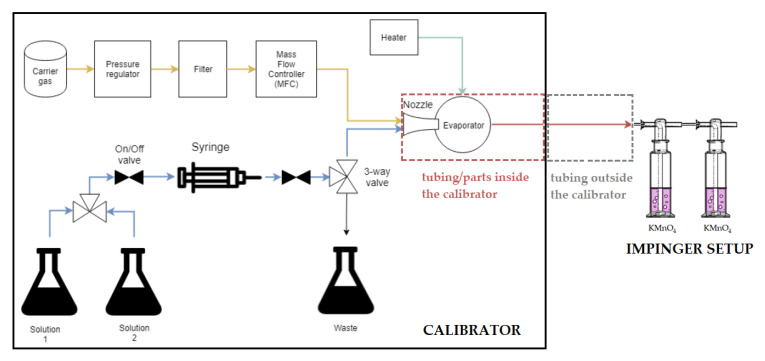
The scheme of the adsorption evaluation setup. Dashed lines show the parts of the setup where the adsorption was evaluated. Adsorption on the tubing/instrument parts inside the calibrator is marked by the red dashed lines, while adsorption on the tubing outside the calibrator is marked by grey dashed lines.

To evaluate any potential adsorption of HgCl_2_ and HgBr_2_ into the calibrator tubing, a two-impinger setup was used, which is a modification of the previously described setup in Figure 2. Since the speciation of the output was not the goal in these experiments, only two consecutive KMnO_4_ impingers (prepared as described above) were used to capture the calibrator output. The permanganate solution retained all the present Hg species [53]; therefore, KCl impingers were not required. The scheme of the complete adsorption evaluation setup is shown in Figure 3.

Since the initial purpose of the calibrator was to be used for HgCl_2_ gas production, more attention was given to this species. To quantify the extent of adsorption, the conditions for capturing the output had to be adjusted. First, the cumulative adsorption of all tubing (inside and outside the calibrator) was evaluated to obtain the whole mass balance. Completely clean tubing inside the calibration unit and outside the calibration unit was used. Without prior preconditioning, the collection of gas output with the two-impinger setup began immediately when the production of HgX_2_ gas started. The collection of output lasted for 20 min. When the collection stopped, the calibrator was immediately switched from the production of HgX_2_ gas to the cleaning protocol (an injection of a blank into the carrier gas–no formation of ^197^HgX_2_ gas) for 1 h to remove the ^197^HgX_2_ adsorbed on the tubing inside the calibrator. The temperature of the calibrator evaporation chamber was 125 °C, and the HgX_2_ gas concentration was 1178 ng m^−3^. The ^197^Hg on the tubing outside the calibrator was cleaned with 45 mL 10% HNO_3_ (*v*/*v*) + 5% HCl (*v*/*v*) acid solution, and 8 mL aliquots were taken for gamma measurement. Eight milliliter aliquots of the impinger solutions were also taken for gamma measurement. Additionally, the ^197^Hg that was adsorbed into the impinger surfaces was thoroughly cleaned with 10 mL of 10% HNO_3_ (*v*/*v*) + 5% HCl (*v*/*v*) acid solution. Eight milliliter aliquots of this washing solution were also taken for gamma measurement.

By skipping the cleaning protocol (an injection of a blank into the carrier gas), we could additionally evaluate the adsorption on the tubing only outside the calibrator. All other experimental parameters were the same as in the paragraph above. Two different temperatures of the calibrator evaporation chamber (125 °C and 180 °C) and two different HgCl_2_ gas concentrations (70.7 and 1178 ng m^−3^) were tested. HgBr_2_ adsorption on tubing outside the calibrator was not evaluated.

## 3. Results and Discussion

To simplify the discussion, the results will for HgCl_2_ gas will be shown first and then the results for HgBr_2_ gas. As the calibrator is intended for HgCl_2_ gas production, most of our efforts were aimed at experiments using HgCl_2_ gas.

### 3.1. Results Using HgCl_2_ Gas

First, we calculated the exact composition of the HgCl_x_^2-x^ species present in the calibrator standard solution. The calculation was done by using the total concentration of chloride ions, the total concentration of mercury, as well as the equilibrium constants for the formation of the HgCl_x_^2-x^ species obtained from the work of Ciavatta and Grimaldi [54]. The full calculation is available in the Supplementary Material. Chloride concentration was always at least three orders of magnitude higher than the total mercury concentration; therefore, mercury concentration did not influence the calculated results. The obtained composition was 0.12% of HgCl^+^, 99.6% of HgCl_2_, and 0.27% of HgCl_3_^−^. Since HgCl_2_ was clearly the most abundant species, we will discuss the results in terms of HgCl_2_. Since water evaporates during the formation of HgCl_2_ calibration gas, the HgCl_2_ concentration changes, and based on the calculation, the relative abundance of HgCl species vastly changes during this process.

#### 3.1.1. Calibrator Time Response Tests

**Figure 4:**
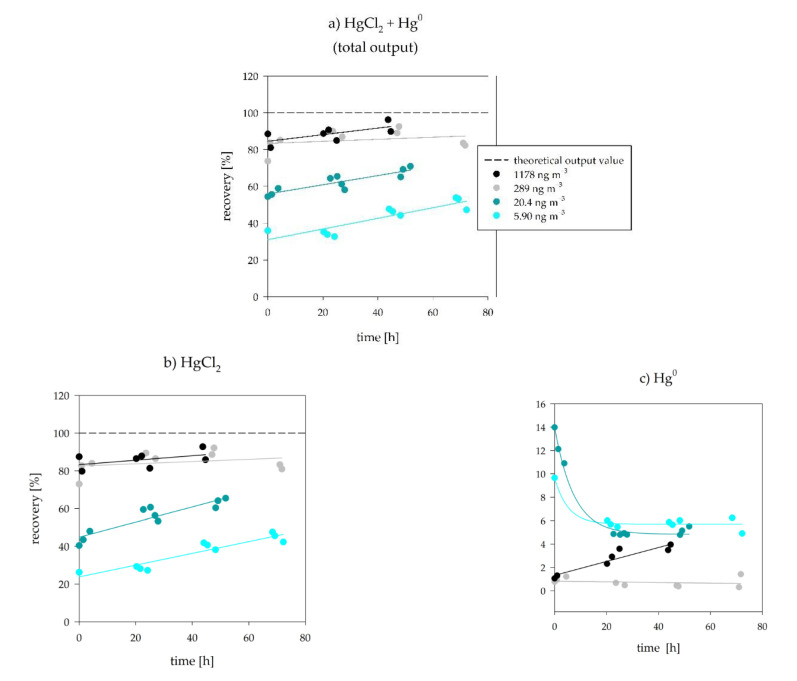
The concentration-dependent response of the HgCl_2_ calibrator output during four days of continuous operation. (**a**) shows HgCl_2_ plus Hg^0^ (total calibrator output), (**b**) shows HgCl_2_, and (**c**) shows the Hg^0^ values. The straight line at 100 percent shows a 100% recovery value, to which the experimentally obtained values were compared. The experiment with 1178 ng m^−3^ concentration had to be finished after 40 h of operation due to a power supply malfunction.

Figure 4 presents the results obtained by the time response tests. Full results are presented in Supplementary Material.

For the 1178 ng m^−3^ HgCl_2_ gas concentration experiments, the initial measurement points started at a recovery of 81% and reached a maximum recovery of 96% after 45 h. For the 289 ng m^−3^ HgCl_2_ gas concentration experiments, the initial measurement points started at a recovery of 74% and reached a maximum recovery of 93% after 48 h (Figure 4a). The absolute percentages of the Hg^0^ present in the calibrator output were between 1.2% and 4.4% for 1178 ng m^−3^ and between 0.3% and 1.7% for 289 ng m^−3^ (Figure 4c). Evidently, the recovery is dependent on the HgCl_2_ gas concentration (Figure 4b). At lower HgCl_2_ gas concentrations, considerably lower recoveries were obtained. For the 20.4 ng m^−3^ HgCl_2_ gas concentration experiments, the initial measurement points started at a recovery of 54% and reached a maximum recovery of 71% after 52 h. For the 5.90 ng m^−3^ HgCl_2_ gas concentration experiments, the initial measurement points started at a recovery value as low as 36% and reached a maximum recovery of 54% after 68 h (Figure 4a). The absolute percentages of the Hg^0^ present in the calibrator output were between 7.3% and 26% for 20.4 ng m^−3^ and between 10% and 27% for 5.90 ng m^−3^ (Figure 4c).

Through all the concentration levels, the cumulative calibrator output (HgCl_2_ + Hg^0^) showed an increase with time since the beginning of calibrator operation. The increase was found to be linear with a similar slope, except for the 289 ng m^−3^ concentration (gray color in Figure 4a). If we were to exclude the last two measurements of the 289 ng m^−3^ experiment, the observed linear slope would be similar to the other gas concentrations. The fraction of Hg^0^ was higher when using low concentrations than when using high concentrations, especially when considering Hg^0^ relative to the whole calibrator output (the relative Hg^0^ values are available in the Supplementary Material). A decrease of Hg^0^ values with time was observed in four out of the five time-trends. The formation of Hg^0^ within the calibrator might have contributed towards the Hg^2+^ + Hg^0^ ⇌ Hg_2_^2+^ comproportionation [55]. Unfortunately, the methods for Hg_2_^2+^ analysis and proof of existence in the calibration solution or in the gas are non-existent for the tested concentration levels. Low recoveries and formation of Hg^0^ might also be due to the lack of check-valves after liquid injection: remaining Hg^2+^ liquid in the tubing could partition to Hg^0^ resulting in losses as shown by the work of Sabri et al. [56]. Even though the findings of Sabri et al. are to be considered, we note that the chemistry of Hg^2+^ in monoethylene glycol is not directly comparable to the chemistry of Hg^2+^ in aqueous solutions.

**Figure 5:**
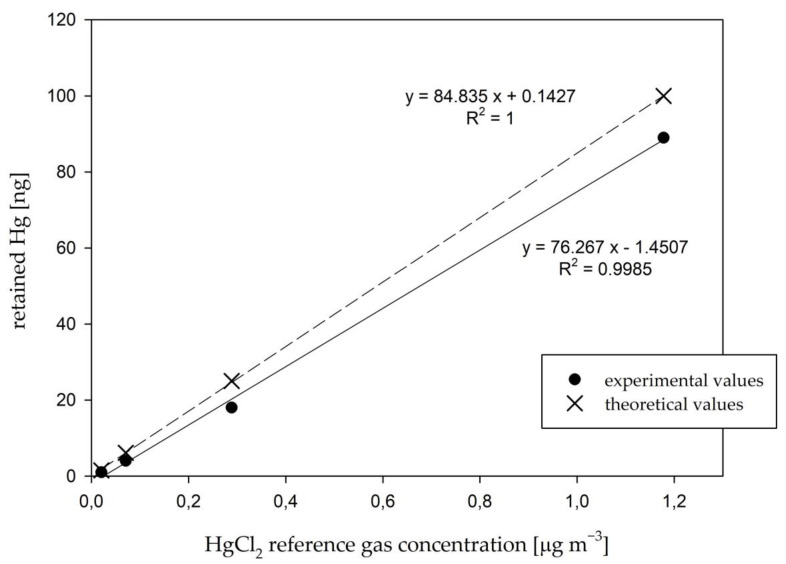
The linearity of the calibrator response at different HgCl_2_ gas concentrations (from 5.90 to 1178 ng m^−3^). Each data point represents a measurement at time 2 h after the calibrator start-up for four different HgCl_2_ gas concentrations. Comparison is shown for theoretical and experimentally determined response.

On the basis of the time response test results, the linearity of the calibrator output was calculated. The results are shown in Figure 5.

While the linear regression resulted in a good correlation coefficient (R^2^ = 0.9985), the slope of the experimental regression (k = 76.267) failed to match the theoretical slope (k = 84.835). Since biased low calibrator outputs were observed in the results above, the discrepancy between two slopes was to be expected. Petrov et al. [39] also observed great linearity of the calibrator output for the ng m^−3^ to µg m^−3^ HgCl_2_ gas concentrations, which is in agreement with the results observed in our work. Even though the difference between the theoretical and experimental slope (Figure 5) might seem minimal, this does not reveal anything about the validity of the calibrator output. As seen previously in Figure 4, recoveries were as low as 32.7% at the beginning of calibrator operation (for the lowest HgCl_2_ gas concentration), which questions the validity of the calibration unit for low HgCl_2_ gas concentrations, even though the linearity of the output is observed. The obtained results illustrate that the linearity of the response by itself is not enough to be certain about the validity of the calibrator output.

It is well known that HgCl_2_ gas tends to adsorb into various surfaces. Therefore, the hypothesis was that consistently low output values could be attributed to the adsorptive nature of HgCl_2_, which would result in losses on the tubing both inside and outside of the calibrator. The results and discussion regarding the stated hypothesis are shown in the next section of the manuscript.

#### 3.1.2. HgCl_2_ Adsorption

First, we performed a duplicate measurement of HgCl_2_ adsorption on combined tubing outside and inside of the calibrator. The experiment is described in detail in Section 2.4. The results are presented in Table 2.

The experiment was performed to show that complete mass balance can be achieved if we combine the HgCl_2_ that reached the two-impinger setup with the adsorbed HgCl_2_ on the tubing. Table 2 shows that the assumption was correct, since a 103% average mass balance was obtained. This was a considerable increase in the mass balance in comparison to the results for the calibrator time response tests in the previous section. The missing percentage of HgCl_2_ was, therefore, attributed to the adsorption of the tubing inside the calibrator. Since the experimental conditions had to be adjusted to enable adsorption measurements, we stress that these results (103% mass balance) are not comparable with the results obtained from the time response experiments (in the previous section). Additional experiments were performed to evaluate the influence of the concentration as well as the calibrator evaporation chamber temperature on HgCl_2_ adsorption. Only the influence of adsorption on the tubing outside the calibrator was evaluated, since it was less time-consuming. The results are presented in Table 3.

The extent of the adsorption varied over different experimental conditions but usually ranged from 15.8% to 18.8%. The exception to this was the experiment with the 125 °C evaporation chamber temperature and the HgCl_2_ gas concentration of 70.7 ng m^−3^; these values ranged from 43.1% to 47.1%, which was considerably higher. These higher values can be attributed to the fact that the lower temperature of the evaporation chamber resulted in a lower gas temperature; consequently, more adsorption on the tubing surfaces occurred. For the 1178 ng m^−3^ HgCl_2_ gas concentration experiment, the effect of the temperature was not as significant.

#### 3.1.3. Efforts to Minimize HgCl_2_ Adsorption

Adsorption was clearly a recurring problem. We tried to solve it in two different ways: with the use of monolithic ceramics and with the saturation of the tubing surfaces with high HgCl_2_ gas concentrations:

(a) Use of monolithic boron nitride for tubing.

For the monolithic ceramic, we tested BN (boron nitride). The monolithic boron nitride (BN) tubing was tested due to the fact that BN is one of the most chemically resistant materials (possibly eliminating chemisorption of HgCl_2_) [57]. Absorption into the Teflon tubing was compared to adsorption into the BN tubing by simply exposing both of these tubes to the same HgCl_2_ gas concentration and then washing the tubing with 10% HNO_3_ (*v*/*v*) + 5% HCl (*v*/*v*) acid solution. Adsorption was still present to an extent, even after using BN; therefore, we concluded that HgCl_2_ adsorption is most probably a combination of chemisorption and physisorption, since BN is known to enhance physisorption [57].

(b) Saturation of adsorption sites.

Using high HgCl_2_ gas concentrations could in a way precondition the system for the use of low HgCl_2_ gas concentration. The active sites on tubing surface would be fully occupied; therefore, HgCl_2_ adsorption could in theory be minimal. The whole experimental procedure was the same as described in Section 2.4.1. The only difference was that for this experiment, a mixture of cold HgCl_2_ (without a ^197^Hg radiotracer) and hot HgCl_2_ (with a ^197^Hg radiotracer) was used as a calibration solution for the formation of HgCl_2_ gas. Cold HgCl_2_ was used solely to saturate the tubing surfaces, while hot HgCl_2_ was used for detection (cold HgCl_2_ was not considered when calculating the recovery). After 24 h, the calibration solution (a combination of cold and hot HgCl_2_) was replaced with hot HgCl_2_ only. Therefore, the gas was switched from the initial high saturating concentration to a low concentration of HgCl_2_.

**Figure 6:**
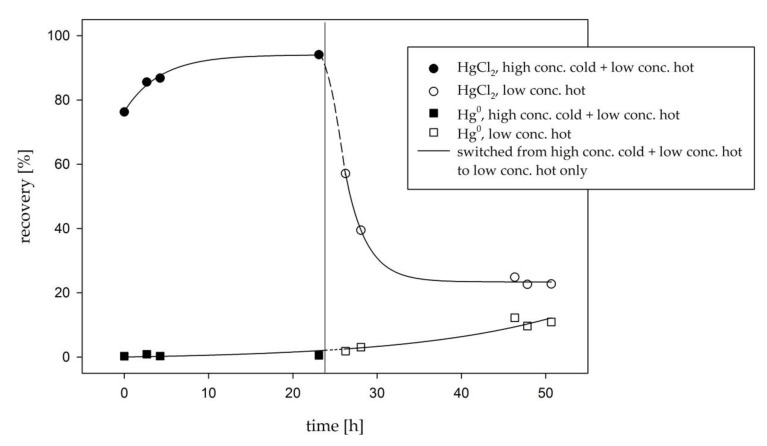
The characterization of the calibrator output when using high HgCl_2_ concentrations (a combination of cold and hot HgCl_2_) and after switching from high to low HgCl_2_ concentrations (hot HgCl_2_ only). The data points labelled “high conc. cold + low conc. hot” were obtained while the gas was comprised of a cold Hg (12.6 µg m^−3^, “saturating” concentration) and a hot Hg (5.90 ng m^−3^, low concentration) mixture. The data points labelled “low conc. hot” were obtained while the gas was comprised of only a hot Hg (low concentration). The continuous lines connecting the data points are estimations of the time trend using different regressions, and the dashed lines are extrapolations of the said regressions.

Recovery values of 100% (calculated as described in previous sections) were compared to the actual calibrator output obtained from the ^197^Hg radiotracer activity. The results of the experiment for behavior of the calibrator under saturating conditions are shown in Figure 6.

The output of the calibrator reached the plateau value (Figure 6) when using a high conc. cold + low conc. hot HgCl_2_. The hypothesis was that after occupying all adsorption places with a high HgCl_2_ concentration, the calibrator output would stay at the same level when switching to a low conc. hot HgCl_2_ only. As the adsorption places would hypothetically stay occupied, that would assure minimal adsorption even when changing to a low HgCl_2_ concentration. Evidently, this was not the case, as the recoveries fell shortly after switching to a low conc. hot HgCl_2_ (Figure 6), meaning that the adsorption of HgCl_2_ was not permanent but collapsed shortly after. Hg^0^ started rising after switching to low HgCl_2_ concentrations. In fact, the recovery values and Hg^0^ fraction approached the values obtained in Figure 4 (5.90 ng m^−3^, light blue data points in the first measurement day).

The clear conclusion was that the method of preconditioning (saturating) the tubing of the setup prior to the use of low HgCl_2_ concentrations in order to lower the effect of adsorption is inefficient. Adsorption in this case proved to be a dynamic process that competed with desorption rather than an irreversible one.

### 3.2. Results Using HgBr_2_ Gas

#### 3.2.1. Calibrator Time Response Tests

Similar to HgCl_2_, we calculated the exact composition of the HgBr_x_^2-x^ species present in the calibrator standard solution. The calculation was performed in the same way as for HgCl_x_^2-x^, the exception being that the equilibrium constants for the formation of the HgBr_x_^2-x^ species were obtained from the work of Hepler and Olofsson [58]. The obtained composition was 98.4% of HgBr_2_ and 1.58% of HgBr_3_^−^. Since HgBr_2_ was clearly the most abundant species, we will discuss the results in terms of HgBr_2_ only. Again, the water evaporates during the formation of HgCl_2_ calibration gas, meaning that the HgBr_2_ concentration changes, and the calculated composition vastly changes during this process.

The time response test results for the HgBr_2_ gas are shown in Table 4.

The calibrator output for HgBr_2_ rose slowly in comparison to the same gas concentrations for HgCl_2_. To compare, the experiment with the 1178 ng m^−3^ HgCl_2_ gas concentration resulted in a maximum recovery value of 96% while for the same concentration, the HgBr_2_ experiment only resulted in a maximum recovery value of 30%. Considering the low starting values of the output, plus the fact that the HgBr_2_ output values increased slowly, the full time-trends for HgBr_2_ were not further investigated. To explain the results, we again proposed the hypothesis that the low recoveries were a result of HgBr_2_ adsorption on the tubing both inside and outside the calibrator.

#### 3.2.2. HgBr_2_ Adsorption on the Outside and Inside Tubing of the Calibrator Combined

A duplicate measurement of HgBr_2_ adsorption on the combined tubing outside and inside the calibrator was made due to the time-consuming nature of the experiment. Mass balances of 100% and 101% were obtained for HgBr_2_. For HgBr_2_, no adsorption experiments for tubing outside the calibrator were made (for HgCl_2_ they were), but since complete mass balance was obtained by the experiment for combined adsorption on tubing outside and inside the calibrator, the presence of such adsorption can be attributed with high certainty.

When comparing HgBr_2_ and HgCl_2_ adsorption, the extent of adsorption seemed to be much greater for HgBr_2_ than for HgCl_2_. The only relevant physicochemical characteristic that separates these two species is their solubility. HgCl_2_ is more soluble in water (6.57 g of HgCl_2_ per 100 mL of water at 20 °C) than HgBr_2_ (0.56 g of HgBr_2_ per 100 mL of water at 20 °C). The dependence of adsorption on solubility is described by the Lundelius rule, which is used to predict (semi-quantitatively) the effect of the chemical character of the solute on its adsorption on surfaces. The rule states that there is an inverse relationship between solute solubility and adsorption, since the solute-solid surface binding competes with the solute-solvent attraction [59].

### 3.3. Thermodynamics of HgCl_2_ Adsorption

Based upon the data obtained in the above described results, the adsorption isotherm was calculated. The previous literature mostly focuses on the adsorption isotherms for HgCl_2_ adsorption on activated carbon [60,61], which is not applicable to our experiments. Therefore, we had to choose the most suitable isotherm for our case (HgCl_2_ adsorption on Teflon tubing surfaces). The Langmuir adsorption isotherm was chosen, as it is most often used to describe chemisorption, which is thought to be the predominant adsorption type at higher HgCl_2_ gas temperatures [62]. Additionally, the Langmuir model resulted in the best fit for our data.

**Figure 7:**
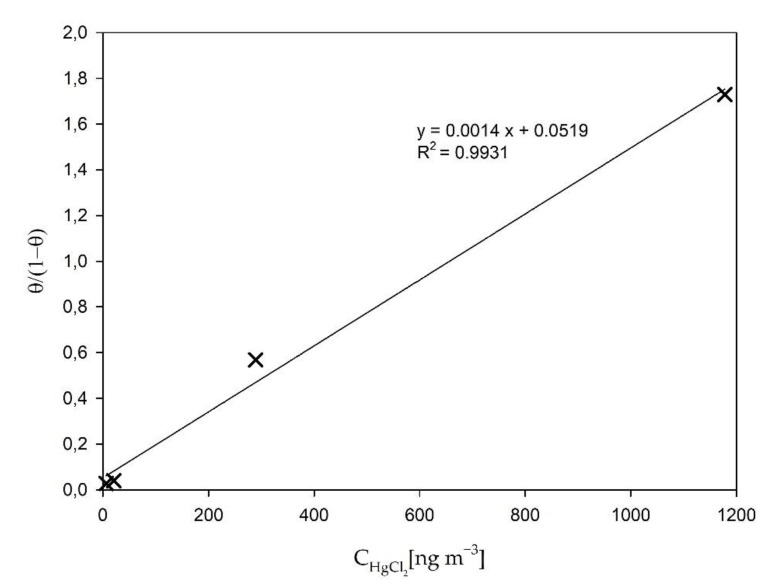
The Langmuir adsorption isotherm for HgCl_2_ adsorption on Teflon tubing: 125 °C. θ presents the occupancy of adsorption sites, while C_HgCl2_ presents HgCl_2_ gas concentration.

The concentration of all available adsorption sites was calculated based on the assumption that for the highest gas concentration time-trend (1178 ng m^−3^), all adsorption sites would be occupied. The concentration of the adsorbed HgCl_2_ was obtained from the adsorption experiments described above. The temperature of the HgCl_2_ gas was assumed to be the same as the temperature of the evaporation chamber (a high gas flow and a short tubing length resulted in minimal cooling of the gas). Figure 7 presents the Langmuir adsorption isotherm for HgCl_2_ adsorption on Teflon tubing at 125 °C.

From the slope (*k*_125°C_ = 0.0014 m^3^ ng^−1^) of the above presented Langmuir isotherm adsorption, the equilibrium constant at 125 °C (*K_eq_*_,125°C_) can be calculated by using Equation (3) [63]:

(3) $$K_{eq}=k\;M_{ads}$$

*M_ads_* is the molecular weight of the adsorbate (in our case HgCl_2_). The Langmuir isotherm was similarly applied for adsorption experiments at 180 °C. From the obtained slope (*k*_180°C_ = 0.0022 m^3^ ng^−1^), the adsorption equilibrium constant at 180 °C (*K_eq_*_,180°C_) was also calculated using Equation (3). From the two temperatures and their corresponding adsorption equilibrium constants, the adsorption enthalpy (Δ*H_ads_*) for HgCl_2_ adsorption on Teflon tubing was calculated using van’t Hoff equation Equation (4) [63]:

(4) $$\frac{d}{dT}lnK_{eq}=\frac{\Delta H^{\theta }}{RT^{2}}$$

The obtained value for adsorption enthalpy was Δ*H_ads_* = −12.33 kJ mol^−1^. The negative value of the enthalpy suggests that the observed adsorption is an exothermal process. Lower temperatures would, therefore, promote adsorption, while higher temperatures would suppress it. This is in line with results observed by other authors [59,63,64].

The Δ*H_ads_* value that was obtained in our work is not an exact value but more likely an estimation, due to the fact that some simplifications (the constant value of gas temperature and the estimation of the number of all adsorption sites) were used in order to be able to obtain Δ*H_ads_*. Additionally, only two data points were available for calculation of Langmuir isotherm at 180 °C, therefore we could only assume that Langmuir isotherm is also best fitting isotherm for 180 °C adsorption from the fact that it was the case for the 125 °C isotherm.

### 3.4. Re-Estimation of the Uncertainty Budget for the Calibration Unit

On the basis of obtained results, the uncertainty budget for the calibration unit was re-estimated in accordance with Guide to the Expression of Uncertainty in Measurement (GUM) [65]. Overall, the uncertainty budget of the Optoseven calibrator was previously estimated to be 2.02% (k = 2) at 10 µg m^−3^ HgCl_2_ gas concentration [33]. By combining 2.02% with the uncertainty component of the recovery values obtained in our work, we calculated the new overall uncertainty values (k = 2), which were 4.10, 5.31, 6.35, and 12.1% for 1178, 289, 20.4, and 5.90 ng m^−3^ gas concentrations, respectively. The equations used for the calculation of the total uncertainty of the calibrator are presented in the Supplementary Material. We acknowledge that the concentration used for the uncertainty budget evaluation by the instrument providers (10 µg m^−3^) is not in the same range as our concentrations. Nevertheless, this was the only data on the uncertainty of the calibration unit that were available to us. Dilutions of the standard solutions (for low gas concentrations) that were performed in addition to what was already considered by the calibrator manufacturers were not included in these calculations, since their contribution was negligible.

Whether or not the calculated uncertainty is acceptable for field measurement is still not clear. It can be concluded that the calibration unit and its uncertainty is suitable for flue gas GOM concentrations (>1 µg m^−3^). The concentration dependence of recoveries prevents the system to be used for calibration of instruments for ambient GOM measurements.

## 4. Conclusions

The ^197^Hg radiotracer has proven to be suitable for studying the characteristics of calibrators because it enabled us to closely follow the processes that limit the use of calibrators based on evaporation, especially for low GOM concentrations. The calibrator could be used for high concentration GOM calibrations, but the recovery and its contribution to the uncertainty must be considered. The recoveries have been shown to be even lower if we replace HgCl_2_ with HgBr_2_, suggesting that different GOM calibration systems will need to be developed (since HgBr_2_ is commonly used as a proxy for all atmospheric HgBr species). In the future, similar calibration units will need to address the adsorption problem in order to provide calibration with ambient GOM levels or, alternatively, to change the principle of operation of the unit. Given that most GOM measurement systems are based on pre-concentration traps/denuders due to low concentrations, it is necessary to develop a calibration system that will be compatible with this pre-concentration method and will have uncertainty that will minimally contribute to the overall uncertainty.

## Figures and Tables

**Table 1:** Comparison of GOM calibration systems.

Authors	Principle of Operation	Species	Concentration Level
Lyman et al. [28]	permeation	HgCl_2_/HgBr_2_	<1 ng m^−3^
McClure et al. [30]		HgBr_2_	1 ng m^−3^
Schaedllich et al. [31] (Hovacal^®^, IAS GmbH)	liquid evaporation	HgCl_2_	N/A, used mostly for > 1 µg m^−3^
Saxholm et al. [33] (Optoseven Ltd. & VTT Ltd.)		HgCl_2_/HgBr_2_ (not yet tested)	>1 µg m^−3^, potential for ng m^−3^ levels

**Table 2:** The adsorption of the tubing inside and outside the calibration unit. A 70.7 ng m^−3^ HgCl_2_ gas concentration and a 125 °C calibrator evaporation chamber temperature were used.

	Tubing Inside the Calibrator [%]	Tubing Outside the Calibrator [%]	Two-Impinger Setup [%]	Sum [%]
replicates	13.2	41.6	46.1	101
	16.3	45.5	43.6	105
average	14.7	43.6	44.8	103

**Table 3:** The HgCl_2_ adsorption of the tubing outside the calibrator in dependence on HgCl_2_ gas concentration and the temperature of the calibrator evaporation chamber.

HgCl_2_ Gas Concentration	Temperature of the Calibrator Evaporation Chamber [°C]	Tubing Outside the Calibrator [%]	Two-Impinger Setup [%]	Sum [%]
70.7 ng m^−3^	125	47.1	43.7	90.7
		43.1	35.4	78.5
	180	18.8	61.8	80.6
1178 ng m^−3^	125	16.9	63.1	80.1
		15.8	76.9	92.8
	180	16.3	74.3	90.6
		17.1	73.6	90.7

**Table 4:** The calibrator output composition during a time-trend experiment using 70.7 and 1178 ng m^−3^ HgBr_2_ gas concentration. Columns “KCl 1,” “KCl 2,” and “KMnO_4_” represent the first KCl impinger (^197^HgBr_2_ retention), the second KCl impinger (^197^HgBr_2_ retention, breakthrough), and KMnO_4_ impinger (^197^Hg^0^ retention), respectively, and are presented as recovery percentages.

HgBr_2_ Gas Concentration	Time [h]	KCl 1 [%]	KCl 2 [%]	KMnO_4_ [%]	Sum [%]
70.7 ng m^−3^	0	9.55	0.05	0.27	9.87
	1.50	10.3	0.42	0.12	10.8
	3.66	11.8	0.07	0.62	12.5
1178 ng m^−3^	0	18.7	3.29	2.01	24.0
	1	27.4	0.07	1.97	29.5
	3	26.3	0.55	2.22	29.1

## Data Availability

Data is contained within the article or Supplementary Material.

## Supplementary Materials

The following are available online at https://www.mdpi.com/article/10.3390/s21072501/s1: Equation S1; Equation S2; Equation S3; Equation S4; Table S1: calibrator output composition during the time-trend experiment using 1178 ng m^−3^ HgCl_2_ gas concentration; Table S2: calibrator output composition during the time-trend experiment using 289 ng m^−3^ HgCl_2_ gas concentration; Table S3: calibrator output composition during the time-trend experiment using 20.4 ng m^−3^ HgCl_2_ gas concentration; and Table S4: calibrator output composition during the time-trend experiment using 5.90 ng m^−3^ HgCl_2_ gas concentration.
