# Supplementary material for: Validating an Evaporative Calibrator for Gaseous Oxidized Mercury
Source: Sensors (Basel). 2021 Apr 3;21(7):2501. doi: 10.3390/s21072501 (PMC8038396; doi:10.3390/s21072501)
Supplement: Supplementary file 1 [file sensors-21-02501-s001.pdf]

Supplementary materials

Validating an evaporative calibrator for gaseous oxidized mercury

Jan Gačnik, [1,2] Igor Živković, [2] Sergio Ribeiro Guevara, [3] Radojko Jaćimović, [2] Jože Kotnik, [2] and Milena Horvat [1,2,] *

[1] Jožef Stefan International Postgraduate School, Jamova cesta 39, 1000 Ljubljana, Slovenia
[2] Jožef Stefan Institute, Department of Environmental Sciences, Jamova cesta 39, 1000 Ljubljana, Slovenia
[3] Laboratorio de Análisis por Activación Neutrónica, Centro Atómico Bariloche, Av. Bustillo km 9.5, 8400 Bariloche, Argentina
* Correspondence: milena.horvat@ijs.si; Tel.: +386-1-588-53-55

Equation S1

$$A_{0,sample} = \frac{A_{sample} * \lambda}{e^{-\lambda * t_{passed}} * [1 - e^{-\lambda * t_{measurement}}]}$$

Equation S2

$$R = \frac{A_{0,sample}}{A_{0,std.}} * \frac{m_{Hg,std.}}{m_{Hg,sample}} * f_{dillution} * 100$$

where:
$A_{0,\ sample}$ is the sample activity at the reference time t=0 [Bq],
$A_{0,\ std}$ is the standard activity at the reference time t=0 [Bq],
A_{sample} is the sample activity at the time of measurement [Bq],
λ is the decay constant [s^{-1}]
$t_{1/2}$ is the half-life of ^{197}Hg [s],
t_{passed} is the time passed since reference time t=0 until the start of measurement [s],
$t_{measurement}$ is the time passed during the measurement [s],
R is the recovery [%],
$m_{Hg,std.}$ is the mass of Hg used for the standard [pg],
$m_{Hg,sample}$ is the mass of Hg used for the sample, assuming 100% recovery [pg],
$f_{dillution}$ is the dilution factor in case the sample and standard were not diluted in the same way.

Equation S1 was applied to calculate both A_0 (activity at reference time) of the sample and A_0 of the standard. The recoveries were calculated using Equation S2.

Equation S3

$$u(R_m) = R_m \times \sqrt{\left(\frac{s_{obs}^2}{n \times C_{obs}^2}\right) + \left(\frac{u(C_{calc})}{C_{calc}}\right)^2}$$

where:
$u(R_m)$ is the uncertainty of the mean recovery,
R_m is the mean recovery,
S_{obs} is the standard deviation of the observed values,
C_{obs} is the observed gas concentration,
n is the number of observed values,
$u(C_{calc})$ is the uncertainty of the calculated (theoretical) gas concentration obtained from the manufacturer,
C_{calc} is the calculated (theoretical) gas concentration.

The standard uncertainty of mean recovery was then incorporated into the combined standard uncertainty by Equation S4. As all other relevant uncertainty components were already included in the calculation of calibrator the uncertainty by the manufacturer; only the uncertainty of the mean recovery had to be added to get the new evaluation of total uncertainty.

Equation S4

$$U = 2\, u_c = 2\sqrt{u_{cal}{}^2 + u_{Rm}{}^2}$$

Where:
U is the expanded uncertainty with a coverage factor k = 2,
u_{cal} is the standard uncertainty of the calibrator obtained from manufacturer,
u_{Rm} is the standard uncertainty of the mean recovery,
u_c is combined standard uncertainty of the calibrator.

Text S1. The calculation of the concentration of $HgCl_x{}^{2-x}$ and $HgBr_x{}^{2-x}$ species present in the calibrator standard solution.

As the calculation was the same for the $HgCl_x{}^{2-x}$ and $HgBr_x{}^{2-x}$ species (the only exception were the values of constants), we will only demonstrate the calculation for the $HgCl_x{}^{2-x}$ species. Four values of equilibrium constants (k) for the formation of the $HgCl_x{}^{2-x}$ species and the complex formation constant (β) were obtained from the literature (also for $HgBr_x{}^{2-x}$) [1, 2].

$$\mathrm{k}_1 = \frac{[\mathrm{HgCl}^+]}{[\mathrm{Hg}^{2+}][\mathrm{Cl}^-]} \qquad \mathrm{k}_2 = \frac{[\mathrm{HgCl}_2]}{[\mathrm{HgCl}^+]\,[\mathrm{Cl}^-]} \qquad \mathrm{k}_3 = \frac{[\mathrm{HgCl}_3{}^-]}{[\mathrm{HgCl}_2][\mathrm{Cl}^-]} \qquad \mathrm{k}_4 = \frac{[\mathrm{HgCl}_4{}^{2-}]}{[\mathrm{HgCl}_3{}^-][\mathrm{Cl}^-]} \qquad \beta = \frac{[HgCl_2{}^{2-}]}{[Hg^{2+}][Cl^-]^4}$$

where:
log k_1 = 6.72, log k_2 = 6.51, log k_3 = 1.00, log k_4 = 0.97, log β = 15.2

The total concentration of Hg ($[Hg_T]$) and Cl ($[Cl_T]$) species was known; therefore, two additional equations were obtained:

$$[Hg_T] = [Hg^{2+}] + [HgCl^+] + [HgCl_2] + [HgCl_3{}^-] + [HgCl_4{}^{2-}]$$

$$[Cl_T] = [HgCl^+] + 2[HgCl_2] + 3[HgCl_3{}^-] + 4[HgCl_4{}^{2-}] + [Cl^-]$$

Since we had 7 equations (equations for k_1, k_2, k_3, k_4, β, $[Hg_T]$ and $[Cl_T]$) and 6 variables ($[Hg^{2+}]$, $[HgCl^+]$, $[HgCl_2]$, $[HgCl_3{}^-]$, $[HgCl_4{}^{2-}]$ and $[Cl^-]$), the analytical solution for this system of equations was obtainable. Using the described system, we could then calculate the concentration of all $HgCl_x{}^{2-x}$ species for each $[Hg_T]$ (which varied over the conducted experiments). As already mentioned in the manuscript, the total chloride concentration exceeded the total mercury concentration by over 3 orders of magnitude; therefore, varying the total Hg concentration did not result in considerably different calculated values of $HgCl_x{}^{2-x}$.

A similar calculation was also performed for the formation of $HgBr_x{}^{2-x}$.

Table S1. The calibrator output composition during the time-trend experiment using 1178 ng m^{-3} $HgCl_2$ gas concentration. Columns "KCl 1," "KCl 2," and "$KMnO_4$" represent the first KCl impinger ($^{197}HgCl_2$ retention), the second KCl impinger ($^{197}HgCl_2$ retention, breakthrough), and $KMnO_4$ impinger ($^{197}Hg^0$ retention), respectively, and are presented in relative terms as a percentage of the whole e mass balance (% of X for convenience).

time passed since calibrator start-up [h]	mass balance, X [%]	KCl 1 [% of X]	KCl 2 [% of X]	$KMnO_4$ [% of X]
0	88.5	97.4	1.41	1.19

1.00	81.0	96.8	1.63	1.60
20.2	88.7	95.4	2.04	2.60
22.1	90.7	95.2	1.61	3.21
25.0	84.9	94.1	1.63	4.23
43.7	96.2	94.6	1.76	3.62
44.7	89.8	93.9	1.69	4.40

Table S2. The calibrator output composition during the time-trend experiment using 289 ng m^{-3} $HgCl_2$ gas concentration. Columns "KCl 1," "KCl 2," and "$KMnO_4$" represent the first KCl impinger ($^{197}HgCl_2$ retention), the second KCl impinger ($^{197}HgCl_2$ retention, breakthrough), and the $KMnO_4$ impinger ($^{197}Hg^0$ retention), respectively, and are presented in relative terms as a percentage of the whole mass balance (% of X for convenience).

time passed since calibrator start-up [h]	mass balance, X [%]	KCl 1 [% of X]	KCl 2 [% of X]	$KMnO_4$ [% of X]
0.0	73.7	98.4	0.57	1.04
0.83	83.6	98.0	0.92	1.11
4.50	85.1	97.8	0.81	1.42
23.6	90.0	98.5	0.76	0.74
27.0	86.9	99.0	0.45	0.54
47.0	89.0	99.1	0.43	0.50
47.7	92.5	99.3	0.29	0.41
71.0	83.5	99.1	0.59	0.36
71.7	82.3	98.0	0.28	1.72

Table S3. The calibrator output composition the during time-trend experiment using 20.4 ng m^{-3} $HgCl_2$ gas concentration. Columns "KCl 1", "KCl 2," and "$KMnO_4$" represent first KCl impinger ($^{197}HgCl_2$ retention), the second KCl impinger ($^{197}HgCl_2$ retention, breakthrough), and the $KMnO_4$ impinger ($^{197}Hg^0$ retention), respectively, and are presented in relative terms as a percentage of the whole mass balance (% of X for convenience).

time passed since calibrator start-up [h]	mass balance, X [%]	KCl 1 [% of X]	KCl 2 [% of X]	$KMnO_4$ [% of X]
0.00	54.4	73.3	0.98	25.7
1.42	55.6	77.5	0.78	21.8
3.75	58.9	80.4	1.06	18.5
22.7	64.3	90.5	1.98	7.56
25.2	65.4	90.8	1.90	7.34
26.8	61.2	90.6	1.35	8.05
27.9	58.1	88.0	3.74	8.27
48.3	65.1	90.8	1.83	7.35
49.1	69.2	90.8	1.75	7.44
51.8	70.9	83.5	8.71	7.77

Table S4. The calibrator output composition during the time-trend experiment using 5.90 ng m^{-3} $HgCl_2$ gas concentration. Columns "KCl 1," "KCl 2," and "$KMnO_4$" represent the first KCl impinger ($^{197}HgCl_2$ retention), the

second KCl impinger ($^{197}HgCl_2$ retention, breakthrough), and the $KMnO_4$ impinger ($^{197}Hg^0$ retention), respectively, and are presented in relative terms as a percentage of the whole mass balance (% of X for convenience).

time passed since calibrator start-up [h]	mass balance, X [%]	KCl 1 [% of X]	KCl 2 [% of X]	$KMnO_4$ [% of X]
0.0	35.9	69.7	3.37	26.9
20.3	35.3	79.2	3.75	17.0
21.6	33.8	79.2	3.99	16.8
24.2	32.7	78.2	5.10	16.7
44.1	47.7	85.5	2.26	12.3
45.4	46.3	85.2	2.62	12.2
48.2	44.2	63.3	3.00	13.6
68.4	53.8	86.9	1.48	11.6
69.2	53.2	84.4	1.32	14.3
72.2	47.2	87.6	2.01	10.4

References:

1. L. G. Hepler and G. Olofsson, “Mercury: Thermodynamic Properties, Chemical Equilibria, and Standard Potentials,” Chem. Rev., vol. 75, no. 5, pp. 585–602, 1975, doi: 10.1021/cr60297a003.
2. L. Ciavatta and M. Grimaldi, “Equilibrium Constants of Mercury (II) Chloride Complexes,” J. Inorg. Nucl. Chem., vol. 30, no. Ii, pp. 197–205, 1968.
